# Water Circulation and Marine Environment in the Antarctic Traced by Speciation of ^129^I and ^127^I

**DOI:** 10.1038/s41598-017-07765-w

**Published:** 2017-08-10

**Authors:** Shan Xing, Xiaolin Hou, Ala Aldahan, Göran Possnert, Keliang Shi, Peng Yi, Weijian Zhou

**Affiliations:** 1 State Key Laboratory of Loess and Quaternary Geology, Shaanxi Key Laboratory of Accelerator Mass Spectrometry Technology and Application, Xi’an AMS Center, Institute of Earth Environment, Chinese Academy of Sciences, Xi’an, 710061 China; 2 Technical University of Denmark, Center for Nuclear Technologies, Risø Campus, Roskilde, 4000 Denmark; 3 Department of Geology, United Arab Emirates University, Al Ain, 17511 United Arab Emirates; 4 Department of Earth Sciences, Uppsala University, Uppsala, 75120 Sweden; 5 Tandem Laboratory, Uppsala University, Uppsala, 75120 Sweden; 6 School of Nuclear Science and Technology, Lanzhou University, Lanzhou, 73000 China; 7 College of Hydrology & Water Resources, Hohai University, Nanjing, 210098 China

## Abstract

Emissions of anthropogenic ^129^I from human nuclear activities are now detected in the surface water of the Antarctic seas. Surface seawater samples from the Drake Passage, Bellingshausen, Amundsen, and Ross Seas were analyzed for total ^129^I and ^127^I, as well as for iodide and iodate of these two isotopes. The variability of ^127^I and ^129^I concentrations and their species (^127^I^−^/^127^IO_3_^−^, ^129^I^−^/^129^IO_3_^−^) suggest limited environmental impact where ((1.15–3.15) × 10^6^ atoms/L for ^129^I concentration and (0.61–1.98) × 10^−11^ for ^129^I/^127^I atomic ratios are the lowest ones compared to the other oceans. The iodine distribution patterns provide useful information on surface water transport and mixing that are vital for better understanding of the Southern Oceans effects on the global climate change. The results indicate multiple spatial interactions between the Antarctic Circumpolar Current (ACC) and Antarctic Peninsula Coastal Current (APCC). These interactions happen in restricted circulation pathways that may partly relate to glacial melting and icebergs transport. Biological activity during the warm season should be one of the key factors controlling the reduction of iodate in the coastal water in the Antarctic.

## Introduction

The Antarctica and the Southern Ocean play profound roles on the response to global climate system. Antarctic Circumpolar Current (ACC), one of major currents of the Southern Ocean, flows from west to east across the Atlantic, Indian and Pacific oceans due to the westerlies circulation and has vital role in the redistribution of marine constituents among these oceans such as water mass, heat and salinity. The spread of anomalous features of this water among those oceans through ACC has a huge effect on regional and even global climate. Meanwhile, due to the global warming, the strengthening of high-altitude meridional temperature gradient between tropic and polar region has strengthened the westerly jet greatly^1, 2^, which may affect the adjustment of local climate of the Southern Oceans and even global climate through possible drift in the thermohaline circulation^3^. The Southern Ocean currents including ACC and Antarctic Peninsula Coastal Current (APCC) have critical influences on the change of configuration and function of ecosystem of the Antarctica^4^. The World Conservation Union has announced in 2016 that the world’s largest marine protected park in the Ross Sea will be the world largest environmentally marine protected reservoir. Therefore, it is vital to trace the regional water gyres and circulations between the ACC and APCC, which are effective transmission channels of heat and nutrients^5^ and intensively affect the ecosystem of the Southern Ocean. Investigation of the sources, transport pathways and exchange of water masses in the Southern Ocean will provide critical information about the mechanisms of marine water change and effects on the environment and ecosystem.

Anthropogenic iodine-129 has been proved to be a valuable oceanographic tracer in investigation of water circulation, particularly in the Arctic^6–12^. Iodine exists predominantly as dissolved iodate, iodide, and a minor amount of organic iodine in the ocean^13^. Although iodide is a thermodynamically unfavorable species in oxygenated water, its formation through the reduction of iodate cannot occur spontaneously by chemical means alone. Iodate, on the other side, is a thermodynamically favorable species of iodine in seawater, but the kinetic barrier prevents the direct oxidation of iodide to iodate^13^. However, some biological processes including bacteria, enzyme and plankton activity have been suggested to control the formation of iodide^14^. Therefore, the transformation of iodine species, and in particular the ^129^I species, can reflect the change of marine primary production and marine environment^14, 15^.

The only stable isotope of iodine is ^127^I, and the most long-lived radioisotope (T_1/2_ = 15.7 My) is ^129^I with a pre-nuclear era natural ratio (^129^I/^127^I) of less than 1.5 × 10^−12^ in the marine system^16^. Human nuclear activities including nuclear weapons test, nuclear fuel reprocessing and nuclear accidents have released a large amount of ^129^I to the Erath’s environments, and elevated ^129^I level by 1–5 orders of magnitude, up to values (^129^I/^127^I) exceeding 10^−7^. Due to uniqueness of ^129^I resources and the relatively long residence time of iodine (~300 ky) compared with the water turnover time (~1000 y) in the ocean^17^, ^129^I is a useful tracer for investigation of ocean circulation and water mass exchange^14, 18–20^. These studies have mainly focused on the Northern Hemisphere, especially the North Atlantic and Arctic, the highest value up to 4 × 10^12^ atoms/L for ^129^I concentration, and 3 × 10^−6^ for ^129^I/^127^I atomic ratio occurred in the English Channel and the North Sea^14^. Only a few data from the Southern Hemisphere (40–75°S) were available^21, 22^, where the lowest value down to (1–3) × 10^6^ atoms/L for ^129^I concentration, and (6–13) × 10^−12^ for ^129^I/^127^I atomic ratio were reported in the Antarctic water^23^.

It is generally known that pollutants are transported to the Antarctic through atmosphere dispersion and ocean currents. We have reported the dispersion and pathway of gaseous pollutant to the Antarctic from Northern Hemisphere and lower latitude region in our previous study^23^. The investigation presented here aims to examine levels and distribution of iodine species (^129^I and ^127^I) in the surface seawater in the Drake Passage, Bellingshausen, Amundsen, Marie Byrd and Ross Seas in the Antarctic sector. The information is used to trace the sources and transport pathways of different species of iodine isotopes in the Antarctic water and to contribute for the better understanding of circulation and movement of the water masses and its effects on the marine environment.

## Results

### Distribution of ^127^I and ^129^I in the Antarctic surface seawater

**Figure 1:**
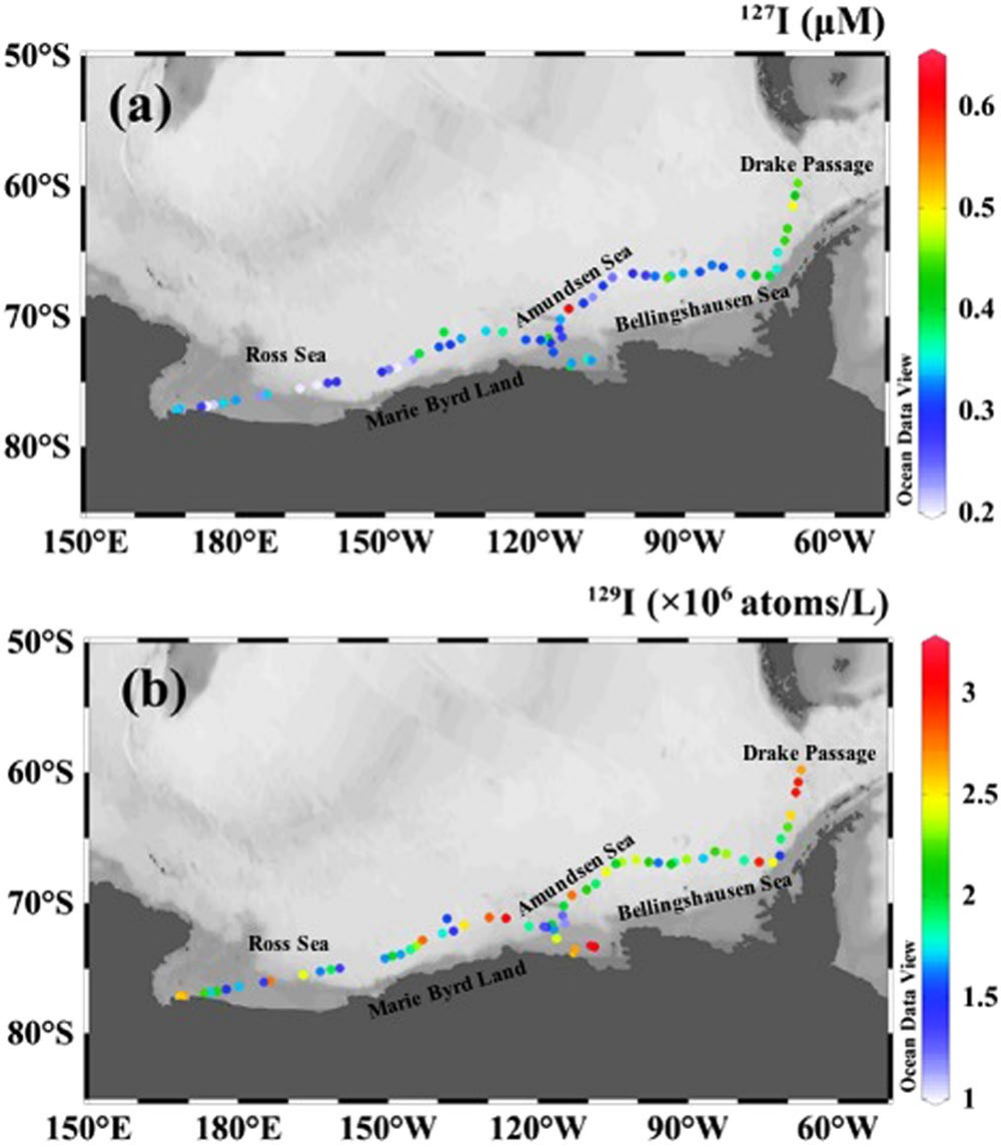
Variation in concentration of total ^127^I and ^129^I in the Antarctic surface seawater. The original map was constructed by a free software Ocean Date View (ODV 4.7.8) (Schlitzer, R., Ocean Data View, odv.awi.de, 2017).

The concentrations of ^127^I in the Antarctic surface waters (Fig. 1a, Supplementary Table S1) range from 0.20 μmol/L to 0.60 μmol/L, with an average of 0.32 μmol/L. The data show big variation in the concentrations along the sampling area. Relatively high ^127^I concentrations (0.35–0.60 μmol/L) occurred in the Drake Passage, central Bellingshausen Sea, central Amundsen Sea and its coastal area, central Marie Byrd and Ross Sea coast. The concentrations of ^129^I (Fig. 1b, Supplementary Table S1) in the analyzed seawater range from 1.15 × 10^6^ atoms/L to 3.15 × 10^6^ atoms/L, with an average of 2.14 × 10^6^ atoms/L, which is lower than that in the Northern Hemisphere (>1.0 × 10^7^ atoms/L)^24^ by a factor of more than 3. As the case with ^127^I, high ^129^I concentrations ((2.5–3.15) × 10^6^ atoms/L) occur in the Drake Passage, eastern Bellingshausen Sea, central Amundsen Sea and its coastal area, eastern Marie Byrd and central Ross Sea. Considerable variability is also observed in the ^129^I/^127^I atomic ratios with values ranging from 0.61 × 10^−11^ to 1.98 × 10^−11^ (Supplementary Table S1, Supplementary Fig. S1). These values are more than 4 times higher than the pre-nuclear level of ^129^I/^127^I in the marine system (1.5 × 10^−12^)^16^.

### Chemical species of ^127^I and ^129^I and their distribution in the Antarctic surface seawater

**Figure 2:**
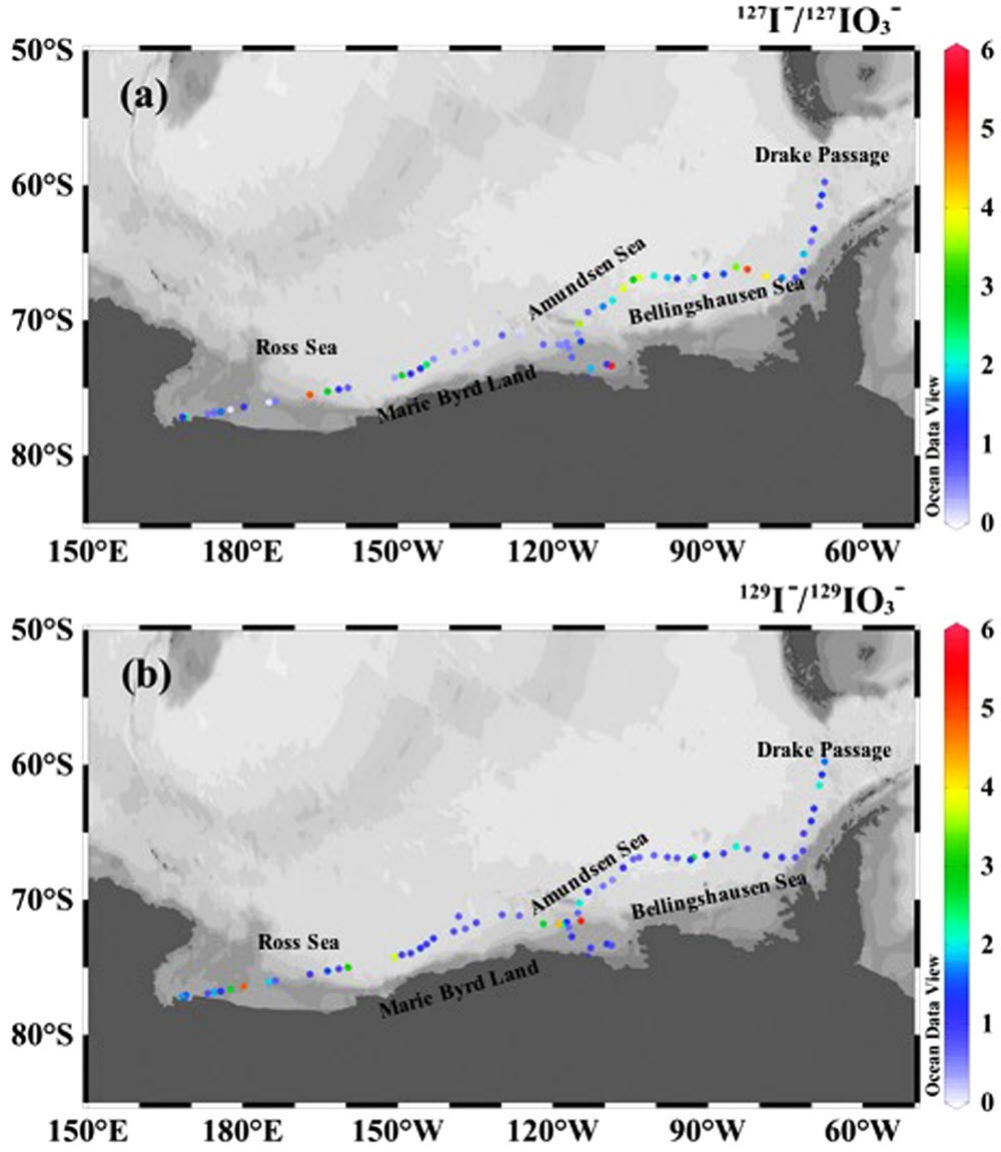
Variation of ^129^I^−^/^129^IO_3_^−^ and ^127^I^−^/^127^IO_3_^−^ molar ratio in Antarctic surface seawater. The original map was constructed by a free software Ocean Date View (ODV 4.7.8) (Schlitzer, R., Ocean Data View, odv.awi.de, 2017).

Distributions of the molar ratios of ^127^I^−^/^127^IO_3_^−^ and ^129^I^−^/^129^IO_3_^−^ (here after will be referred to as ratio) in the Antarctic surface waters are shown in Fig. 2 and Supplementary Table S2, and the values range at 0.03–5.85 for ^127^I^−^/^127^IO^3−^ with an average of 1.44 and at 0.44–5.19 for ^129^I^−^/^129^IO_3_^−^ with an average of 1.37. The ^129^I^−^/^129^IO_3_^−^ ratios in the Drake Passage (locations 1, 3, 5), central and western Amundsen Sea (locations 25, 37, 38, 39) and central and western Ross Sea (locations 57,59,61) are more than 1, while ^127^I^−^/^127^IO_3_^−^ ratios are less than 1 indicating that ^129^I and ^127^I exist predominantly as iodide and iodate, respectively. On the contrary, ^127^I^−^/^127^IO_3_^−^ ratios in the Bellingshausen Sea (locations 6,7,9–11, 13, 17, 18), eastern Amundsen Sea (locations 19–21,23,24), western Marie Byrd (locations 47, 49, 50) and eastern Ross Sea (locations 53, 55) are higher than 1, while the ^129^I^−^/^129^IO_3_^−^ ratios are less than 1. This indicates that ^127^I and ^129^I exist predominantly as iodide and iodate, respectively. The ^129^I^−^/^129^IO^3−^ and ^127^I^−^/^127^IO_3_^−^ ratios in the eastern Marie Byrd (locations 40–46) are less than 1, which is slightly higher than the reported ^127^I^−^/^127^IO_3_^−^ molar ratios of surface seawater in the Drake Passage (0.06–0.24) and Weddell Sea (0.04–0.12)^25^, suggesting a predominant species of ^127^I and ^129^I is iodate in this area. The ^129^I^−^/^129^IO_3_^−^ molar ratios of (0.79–1.72) and ^127^I^−^/^127^IO_3_^−^ of (0.76–5.85) in the coastal water of the Amundsen Sea and Ross Sea are similar to those observed in the coastal areas of the North Sea^14^.

## Discussion

**Figure 3:**
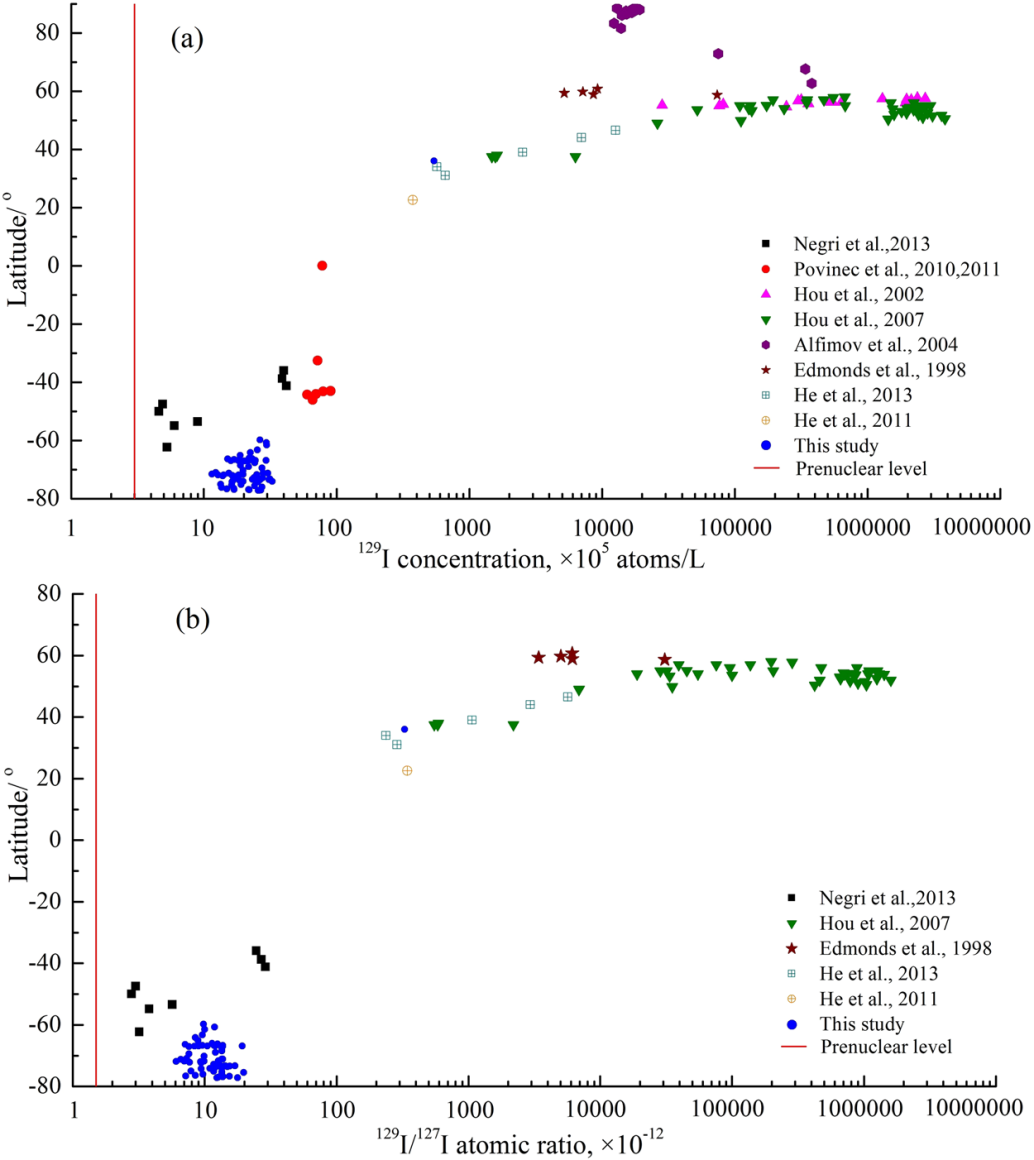
Comparison of ^129^I concentrations (**a**) and ^129^I/^127^I atomic ratios (**b**) in Antarctic seawater with those in other marine waters. The red line indicates pre-anthropogenic values. The data of five locations (7, 37, 42, 45 and 51) have been reported in the Ref. 23.

The concentrations of ^129^I and the ^129^I/^127^I ratios in the Antarctic surfaces seawater show the lowest values compared to those measured in surface waters of other oceans (Fig. 3). The ^129^I concentrations in the Antarctic surface seawater are 3–5 orders of magnitude lower than what was found in the surface water of the Nordic Seas and the Arctic Ocean^7, 14, 20, 26^. Sources of the considerably high ^129^I in the North Atlantic and the Arctic Oceans and related seas were attributed to dispersion of marine discharges from the nuclear reprocessing plants at La Hague (France) and Sellafield (UK). Even the relatively low ^129^I values ((0.6–0.9) × 10^7^ atoms/L) measured in seawaters in the Indian Ocean^22^ are 2–8 times higher than those in the Antarctic. The only reported values of ^129^I concentrations ((0.5–0.9) × 10^6^ atoms/L) and ^129^I/^127^I ratio ((0.3–0.6) × 10^−11^) in the shallow seawater, which are slightly lower than those in Antarctic surface seawater (59–77°S) measured here, were observed in the southern South Pacific Ocean (47–62°S)^21^. The low ^129^I concentrations in the Pacific Ocean waters might result from an underestimation of ^129^I concentration because organic ^129^I was not separated using solvent extraction and excluded in the measured ^129^I^27^. In addition, the ^127^I concentrations in those seawater samples collected in the shallow are much lower (30−35 μg/L) than that in open sea water (about 60 μg/L), indicating a possible dilution of ^129^I by freshwater/ground waters with low ^129^I concentration.

Irrespective of the ^129^I concentration variability in the world’s oceans, all the data shown in Fig. 3 indicate values above the natural concentration in the pre-anthropogenic era seawater. Fallout from nuclear weapons tests, discharges from Nuclear fuel reprocessing plants (NFRPs) and nuclear accidents such as at Chernobyl and Fukushima are the major anthropogenic sources of ^129^I to the environment. Each of these sources has dispersed in the Earth’s surface environment through particular spatial and temporal mode. Dispersion in the atmosphere was the main mode of the nuclear weapons testing derived radioactive releases, while the main pathway of the NFRPs releases is through marine waters. Both of these sources have globally spatial and a few decades temporal extension compared to the restricted regional effects of the nuclear accidents. The NFRPs in the Southern Hemisphere are the ones in Argentina, Brazil and South Africa. Antarctica marine waters lie far from any direct discharges from these NFRPs and the nuclear weapons testing sites. Consequently, it is expected that ^129^I in the Antarctica marine waters originates from remote sources and was transported through ocean currents and/or atmospheric dispersion.

^129^I released from nuclear weapons testing under seawater may directly enters the seawater, while the atmospheric fallout contributes to ^129^I produced by atmospheric weapons testing, including the close-in (tropospheric) fallout for tests conducted in small islands in the Pacific Ocean. Some of the atmospheric nuclear weapons tests conducted at the Pacific Proving Grounds (PPG) mainly in the Marshall Islands in 1946–1962, yielded about 108.5 Mt TNT, corresponding to about 24.7% of the total yield of global nuclear weapons tests^28^. Close-in fallout of the PPG was preferentially deposited in the sea since the tests were conducted on small islands. It has been demonstrated that close-in fallout of radionuclides (e.g. plutonium) from the PPG has been transported to the northwestern Pacific Ocean by the North Equatorial Current and Kuroshio Current^29, 30^. There is no evidence, however, indicating that the radionuclides of close-in fallout from the PPG were transported through sea currents to the lower latitude region of the Northern Hemisphere and across the equator to the Southern Hemisphere.

Pointing out a major source of ^129^I to the Antarctica marine water has critical used to investigate water circulation and glacier balance. If the major source is through the marine circulation, then it can elucidate where this happening is and how this can be connected to water circulation. Alternatively, if the source is dominated by atmospheric dispersal, then much of iodine can also be trapped in the ice cap and can provide indications of glacier contribution to the marine waters. Transport of the ^129^I released from NFRPs which accounts to more than 90% of the ^129^I in the present environment, mainly occurs via marine circulation and less restricted dispersion via the atmosphere^6, 26^. These dispersion patterns have resulted in a dramatically elevated ^129^I level in the Northern North Atlantic and the Arctic Oceans by 2–4 orders of magnitude, with reported ^129^I/^127^I atomic ratios up to 10^−6^ in the North Sea and 10^−8^ in the Arctic^14^. Some investigations reported possible dispersal of the European NFRPs derived ^129^I to Asia and North America through atmosphere^31, 32^. While no evidence shows that the European NFRPs derived ^129^I has reached the Antarctica waters through the atmospheric dispersion and fallout^23, 33^.

A few atmospheric nuclear weapons tests were conducted in the South hemisphere, including the French tests at Mururoa (22.2°S) and Fangatau Atolls (15.8°S) in 1966–1974 with total yield of 10.2 Mt TNT, corresponding to about 2.32% of the total yield of all atmospheric nuclear weapons testing^28^, and British tests at Christmas Island (10.5°S), Monte Bello Islands (20.4°S), and Maralinga and Woomera in Australia (29–31°S) in 1957–1958 with yield of 8.05 Mt TNT corresponding to about 1.83% of the total yield^28^. It has been estimated that an approximate rate of 0.17 and 0.28 g of ^129^I per kiloton TNT equivalent is produced from fission of ^235^U and ^239^Pu, respectively in a nuclear explosion. It is estimated that the French and British nuclear weapon tests have released a total of 2.1–5.1 kg ^129^I, which accounts for about 5% of the total ^129^I (~57 kg)^34^ released from the global atmospheric nuclear weapons tests. Therefore, the ^129^I released from these weapons tests is an important source of ^129^I in the Southern Hemisphere seawaters.

^129^I in the Antarctic surface seawater should also originate from remote areas through atmospheric dispersion in the stratosphere. Occurrence of tritium, ^137^Cs, ^36^Cl, ^90^Sr,^238, 239, 240, 241^Pu and^241^Am in the ice cores and snow in the Antarctica was attributed to nuclear weapons testing^35–38^, although fallout levels of these radionuclides are more than 2 orders of magnitude lower than those observed in the Northern Hemisphere. Time series records of the radionuclides in the ice cores indicated two peaks, corresponding to the nuclear weapons tests in the Northern Hemisphere in the early 1950s and 1960s. The tests include the ones conducted at the PPG in Marshall Islands in low latitude region of the North Pacific Ocean in 1952–1954 (Ivy test, November 1952 and Castles test, March 1954, respectively), and those in 1961–1962 in the middle and high latitude region of the Northern Hemisphere (Nevada, Semipalatinsk and Novaya Zemlya). However, unlike the deposition model in the Northern Hemisphere, the highest deposition of ^90^Sr and plutonium isotopes (^239,240^Pu) in the Antarctica occurred in 1952–1954 as a result of the stratospheric fallout of weapons tests of the PPG^35, 38^. Although no seawater samples were collected at that time for analysis of ^129^I, it is expected that like other radionuclides mentioned above, ^129^I has also been dispersed through the stratosphere and deposited in the Antarctic. A possible evidence supporting direct influence of atmospheric fallout of the nuclear weapons tests is the ^129^I concentrations ((6–8) × 10^6^ atoms/L and 3.4 × 10^6^ atoms/L) in seawater of the southern Indian Ocean (43–46°S)^22^ and in rivers and lakes of New Zealand (35–45°S)^19^ respectively. These values are higher than the average concentrations measured in Antarctic surface seawater presented here and also higher than ^129^I concentrations ((0.53–0.90) × 10^6^ atoms/L) in seawater in the South Atlantic Ocean (45–62°S)^21^. This feature suggests that much of the ^129^I in Antarctic surface seawater originates from fallout of atmospheric nuclear weapons tests in the Marshall Islands in 1950s through ocean currents transport along the South Pacific.

**Figure 4:**
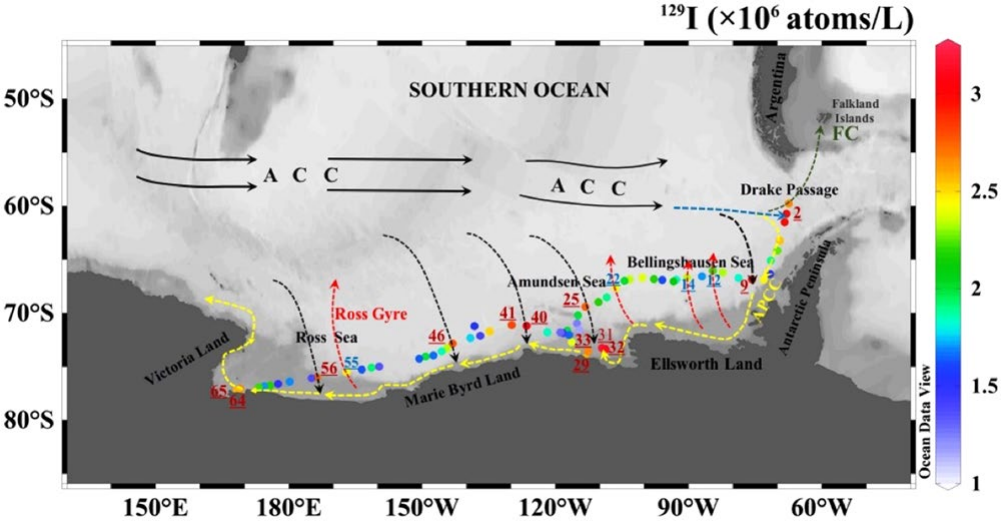
Distribution of ^129^I (10^6^ atoms/L) along the transect suggested surface sea current pathways (dashed arrows). Black dashed arrows for the southern branch of Antarctic Circumpolar Current (ACC); red dashed arrows for the northern branch of Antarctic Peninsula Coastal Current (APCC); yellow dashed arrows for APCC; green dashed arrows for Falkland Current (FC)^49, 50^; black arrows for ACC. The original map was constructed by a free software Ocean Date View (ODV 4.7.8) (Schlitzer, R., Ocean Data View, odv.awi.de, 2017).

Utilization of the isotope concentration to interpret water circulation and environmental impact relies on the characteristics of the system in Antarctic waters (Fig. 4). A major control on the surface water circulation is related to the Antarctic Circumpolar Current (ACC) which is driven by large-scale diagonal compression field moving always eastwards and migrates to the Drake Passage^39^. The ACC interacts with the Antarctic Peninsula Coastal Current (APCC) that generally brings fresher water partly fed by glacial melting. The signatures of this interaction are marked by decrease in ^129^I concentrations from >2.6 × 10^6^ atoms/L at locations 1, 2 and 3 to 1.5 × 10^6^ atoms/L at location 7 in Bellingshausen Sea. This distribution pattern of ^129^I in the surface water of the Drake Passage (Fig. 4) starting with the high ^129^I value is in agreement with the circulation pattern in the region where the ACC converges southwards along the Drake Passage to form the Antarctic Peninsula Coastal Current (APCC) into the Bellingshausen Sea. The decreased ^129^I concentration should be attributed to the dilution of relative high ^129^I in the ACC by the low ^129^I water in the Antarctic Peninsula coast.

The concentrations of ^127^I (0.41 μmol/L) and ^129^I (2.95 × 10^6^ atoms/L) from location 9 in the eastern Bellingshausen Sea are similar to those from location 2 in the Drake Passage. This similarity indicates that an important branch of ACC that carried the high ^127^I and ^129^I migrates southward into the Bellingshausen Sea through location 9. This is also confirmed by the comparable ratios of ^127^I^−^/^127^IO_3_^−^ (1.63) and ^129^I^−^I^−^/^129^IO_3_^−^ (0.97) observed at location 9 with those of ^127^I^−^/^127^IO_3_^−^ (1.34) and ^129^I^−^/^129^IO_3_^−^ (1.32) at location 2. Through weakening of radial stress and strengthening of polar easterlies^40^, the branch of ACC at location 9 is combined with the APCC at location 7, forming a pathway that continues westwards along the Antarctic continent, causing a relative highly ^129^I level in the APCC waters.

It has been proposed that there are a large number of different scales gyres between the ACC and the APCC (i.e. Antarctic Divergence), which are more complicated and contain numerous discontinuous and closed/unclosed gyres^41^. The relatively high ^129^I concentrations ((2.2–2.4) × 10^6^ atoms/L) at locations 12, 14 and 22 suggest that three branches of APCC water with relative high ^129^I level move northward through these locations. This postulation is supported by the relatively high ratios of ^127^I^−^/^127^IO_3_^−^ (1.26–3.76) and ^129^I^−^/^129^IO_3_^−^ (1.11–2.02) at locations 12, 14 and 22 that were caused by the reducing circumstance formed during the high biological activity along the APCC.

The concentrations of iodine isotopes (0.36 μ mol/L for ^127^I and 2.75 × 10^6^ atoms/L for ^129^I) at location 25 are in good agreement with those at location 2 in the Drake Passage and location 9 in the Bellingshausen Sea. This feature might indicate that another branch of the ACC, with the high concentration of iodine isotopes, is driven by the radial wind stress and moves southward across this sampling site^42^. The branched current further moves southward and merges with the APCC, causing the high ^129^I concentration ((2.56–3.15) × 10^6^ atoms/L) observed at locations 29, 31, 32, 33. The high ratios of ^127^I^−^/^127^IO_3_^−^ (0.76–5.85) and ^129^I^−^/^129^IO_3_^−^ (0.79–1.21) observed in this area could be attributed to the anoxic/reducing conditions formed by high biological activities as indicated by the measured high chlorophyll concentration in seawater at location 33 (26.9 μg/L) (Supplementary Table S3, Supplementary Fig. S2).

Relatively high ^129^I concentration of about 2.8 × 10^6^ atoms/L was measured at locations 40, 41 and 46, suggesting additional two branches of ACC that carried the high ^129^I ACC water moving southward across these sampling sites. Relatively low ratios of ^127^I^−^/^127^IO_3_^−^ (0.15–0.68) and ^129^I^−^/^129^IO_3_^−^ (0.58–0.96) were observed in this area compared to those in locations 2 and 3 in the ACC and the APCC. This feature should result from the oxidation of iodide during its transport to this location before emerging with the APCC.

^129^I concentration at location 55 (2.41 × 10^6^ atoms/L) is nearly 2 times higher than that at locations 47–54 (1.34 × 10^6^ atoms/L) and the iodine at this site is mainly iodide (4.85 for ^127^I^−^/^127^IO_3_^−^ and 1.00 for ^129^I^−^/^129^IO_3_^−^). This trend indicates that there is another branch of APCC moving northward through location 55 (167.01°W) towards the ACC. The southward moving ACC branch through location 46 forms a closed gyre (Ross Gyre) with the northward moving branch of the APCC through location 55 (167.01°W). The Ross Gyre was actually one of the first discovered subpolar cyclonic vorticity^43, 44^.

A ^129^I concentration of 2.75 × 10^6^ atoms/L was measured at location 56 (143.10°W), which is comparable to those observed at locations 2, 9, 25, 40–41, 46, indicating another branch of ACC through this location. Low ratios of ^129^I^−^/^129^IO_3_^−^ (0.72) and ^127^I^−^/^127^IO_3_^−^ (0.53) were measured at this site. However, the chlorophyll concentrations at locations 51–54 (east of location 56) and 58–65 (west of location 56) (5.45–18.70) μg/L and (1.66–2.76) μg/L were relatively high. This indicates that existence of iodine species primarily as iodide is due to the phytoplankton activity in both areas (Supplementary Fig. S2).

Relatively high concentrations of ^129^I ((2.57–2.61) × 10^6^ atoms/L) were also observed at locations 64 and 65, indicating the APCC received the high ^129^I seawater from the ACC branches reached to these locations in west bottom of the Ross Sea. This transport pathway is confirmed by the high ^129^I^−^/^129^IO_3_^−^ ratio (1.72) at location 65, which agrees with the observed ^129^I species and the high biological activity in the APCC.

This investigation revealed that the eastward flowing ACC has 6 southward moving branches at the 67.96°W, 75.71°W, 113.19°W, 126.45°W, 143.1°W and 173.7°W, respectively. This non-zonal behavior might be mainly attributed to the complicated submarine topography^45^. Meanwhile, it also confirmed here that the ACC moves eastward along the Pacific-Antarctic ridge to the Drake Passage in the South Pacific and then split into three branches. One branch moves away from the Drake Passage northwards, the second moves eastwards and the third one moves toward the south along the Antarctic Peninsula coast driven by the polar easterlies, and then moves westwards along the Antarctic continent, forming the APCC. Four branches of the APCC move north toward to the ACC. The eastwards moving ACC and westwards moving APCC interact with each other through their branches in opposite directions, forming numerous discontinuous and closed and/or unclosed gyres in-between them.

## Materials and Methods

### Samples and chemicals

**Figure 5:**
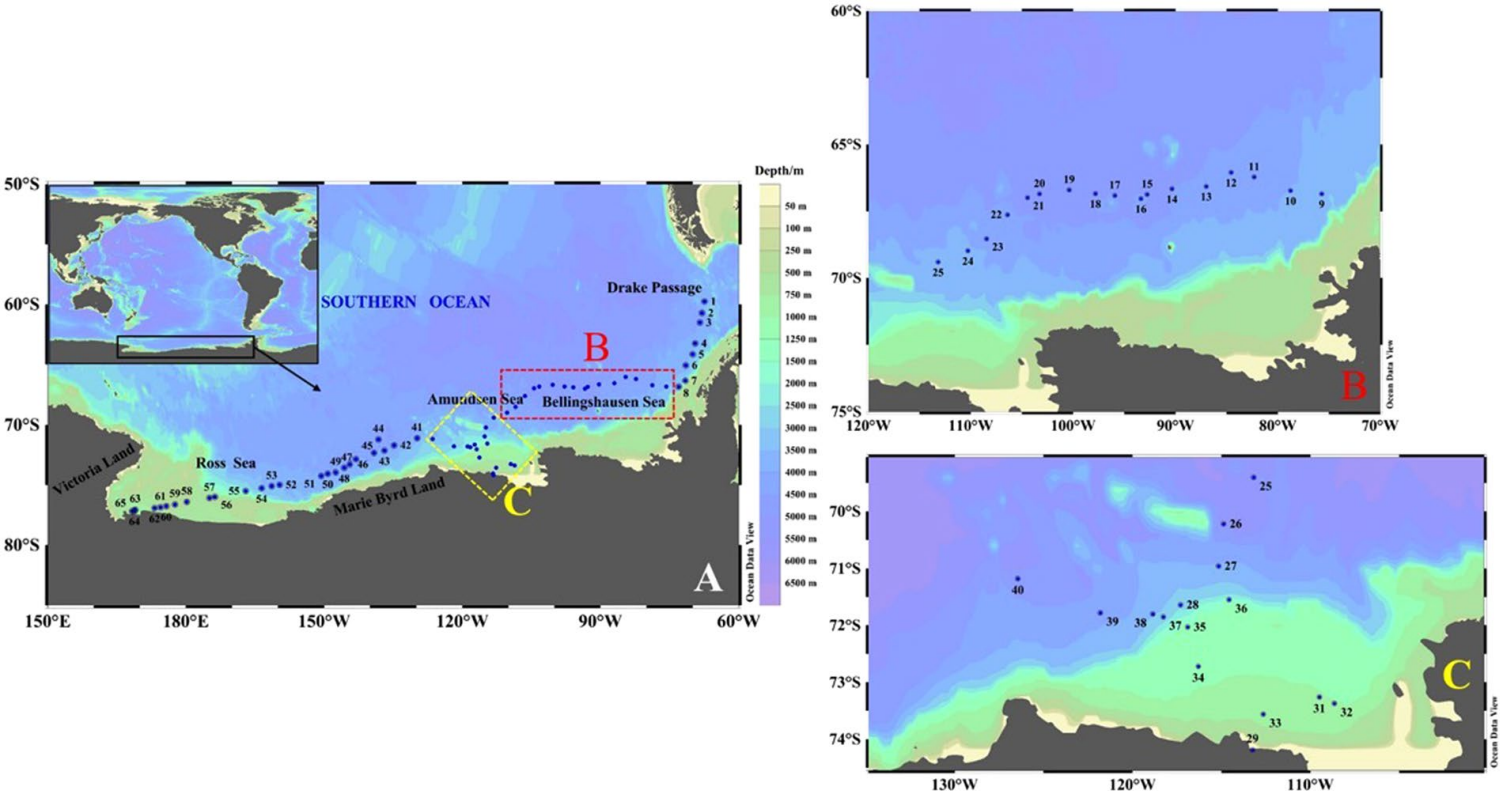
Sampling transect along the Antarctic coast, sampling locations are shown as blue dots. Details of sampling locations of surface seawater in the Bellingshausen Sea (**B**) and in the Amundsen Sea (**C**) are shown in figures 5B and 5C. The original map was constructed by a free software Ocean Date View (ODV 4.7.8) (Schlitzer, R., Ocean Data View, odv.awi.de, 2017).

Sixty-four surface seawater samples were collected in the Drake Passage, Bellingshausen, Amundsen, Marie Byrd and Ross Seas during cruise in the Antarctic (Fig. 5, Supplementary Table S3). The surface seawater was pumped through the built-in seawater sampler in the research vessel N.B. Palmer from 2 m under the sea surface directly into 2 L polyethylene bottles, which were sealed and shipped to Xi’an, China for analysis. Meanwhile, temperature, salinity and chlorophyll concentrations and partial pressure of CO_2_ (pCO_2_) were measured by on-line detecting system in the research vessel.

The ^129^I standard solution (NIST-SRM-4949c, National Institute Standard and Technology Gaithersburg, MD) and ^127^I carrier solution (Woodward iodine, Woodward Iodine Corporation, Oklahoma, U.S.A.) prepared by dissolution of iodine crystal into 0.40 mol/L NaOH-0.05 mol/L NaHSO_3_ solution were used. All chemical reagents used were of analytical grade, and all solutions were prepared using deionized water (18.2 MΩ·cm).

### Separation of total iodine and its species (iodide and iodate) from seawater

After removal of potential suspended particles in the seawater by filtration through a 0.45 μm membrane, 0.60 L and 1.20 L of seawater samples were taken for separation of total iodine and iodine species, respectively. A modified procedure from our previous method was used for separation of total inorganic iodine, iodide and iodate using AgI–AgCl coprecipitation from seawater^46, 47^. In brief, 0.60 L seawater was transferred to a beaker. 0.5 kBq of ^125^IO_3_^−^ tracer was spiked, 0.20 mg of ^127^I carrier and 0.50 ml of 2.0 mol/L NaHSO_3_ solution were added into the beaker, and then 3 mol/L HNO_3_ was added to adjust pH 1–2 to convert all iodine species to iodide. 30 mg Ag^+^ (28 ml of 0.01 mol/L AgNO_3_ solution) was dropwise added to the solution under stirring to form AgI-AgCl-Ag_2_SO_3_-AgBr coprecipitate. The precipitate was separated by centrifuge, and then sequentially washed with 3 mol/L HNO_3_, H_2_O, 30% and 20% NH_4_OH to remove Ag_2_SO_3_ and most of AgCl and AgBr until 1–3 mg of precipitate was obtained. 1.20 L seawater was transferred to a beaker for separation of iodide in seawater. 0.5 kBq of ^125^I^−^ tracer and 0.2 mg of ^127^I^−^ carrier (KI, ^129^I/^127^I atomic ratio <2.0 ± 0.3 × 10^−13^ carrier) were spiked, NaHSO_3_ was added into the sample to a final concentration of 0.30 mmol/L, and then 0.5 mol/L HNO_3_ was slowly added under stirring to adjust pH 4.2–5.5 (measured using a pH meter). 150 mg Ag^+^ (45 ml of 0.03 mol/L AgNO_3_) was dropwise added to the solution to form AgI-AgCl-Ag_2_SO_3_-AgBr coprecipitate. The precipitate was separated by centrifuge and the supernatant was used for separation of iodate. The separated precipitate was sequentially washed with HNO_3_, H_2_O and NH_4_OH until 1–3 mg of precipitate were obtained. To the supernatant, 0.5 kBq^125^IO_3_^−^ tracer was spiked for separation of iodate, 0.2 mg of ^127^I carrier, 0.5 ml of 2.0 mol/L NaHSO_3_ solution were added, and then 3.0 mol/L HNO_3_ was added to adjust pH 1–2 to convert all iodine species to iodide. Then follow the procedure for total inorganic iodine to separate iodate.^125^I in the precipitate was measured using a NaI gamma detector (Model FJ-2021, Xi’an Nuclear Instrument factory, Xi’an, China) for monitoring of the chemical yield of iodine in the procedure. The recovery of iodine and its species in the entire procedure is higher than 80%. The schematic diagram of the analytical procedure is shown in Supplementary Figure S3.

The procedure blanks were prepared using the same procedures as for separation of total iodine, iodide and iodate in seawater but no samples were added.

^129^I/^127^I standards containing 1.0 mg iodine in AgI−AgCl form were prepared for calibration of the AMS measurement. See the detailed method in the Supporting Information. Iodine in the commercial^125^I tracer exists as iodide (NaI). To synthesize^125^IO_3_^−^ tracer, NaClO was added into^125^I^−^ solution, then HCl was added to adjust pH 1–2 to oxidize iodide to iodate. The detailed method is presented in the Supporting Information.

### Measurement of ^129^I using AMS and ^127^I using ICP-MS

The prepared AgI-AgCl coprecipitate was dried in an oven at 60–70 °C for 3–6 h, the dried precipitate was ground to fine powder and mixed with five times by mass of niobium powder (325 mesh, Alfa Aesar, Ward Hill, MA), which was finally pressed into a copper holder using a pneumatic press (Zhenjiang Aode Presser Instruments Ltd.). ^129^I/^127^I atomic ratios in the prepared targets were measured by AMS using 3MV Tandem AMS system (HVEE) in the Xi’an AMS center. All samples, blanks and standards were measured for 6 cycles and 5 minutes per sample in each cycle. A detailed description of AMS system and measurement of ^129^I has been reported elsewhere^48^.

1.0 mL solution of the iodide fraction and the iodate fraction separated using anion exchange chromatography (See the detailed method in the Supporting Information.) and the original seawater were taken to a vial, and the samples were diluted for 10 times using 1% NH_4_OH solution, Cs+ solution was spiked to a concentration of 2 ng/mL. ^127^I in the prepared samples was measured using ICP-MS (X-series II, Thermo Scientific, USA). The detection limit of 0.02 ng/mL for ^127^I was obtained. Iodide concentration was corrected for chemical yield during chromatographic separation.

### Data availability statement

All data analyzed during this study are included in this published article and its Supplementary Information files.

## Electronic supplementary material

supplementary information
