# Supplementary material for: Water Circulation and Marine Environment in the Antarctic Traced by Speciation of 129I and 127I
Source: Sci Rep. 2017 Aug 10;7:7726. doi: 10.1038/s41598-017-07765-w (PMC5552787; doi:10.1038/s41598-017-07765-w)
Supplement: Supplementary file 1 — supplementary information [file 41598_2017_7765_MOESM1_ESM.doc]

**Water Circulation and Marine Environment in the Antarctic Traced by Speciation of ^129^I and ^127^I**

**Shan Xing ^1^, Xiaolin Hou ^1, 2^, Ala Aldahan ^3, 4^, Göran Possnert ^5^, Keliang Shi ^2, 6^, Peng Yi ^4, 7^ & Weijian Zhou ^1^**

^1^ State Key Laboratory of Loess and Quaternary Geology, Shaanxi Key Laboratory of Accelerator Mass Spectrometry Technology and Application, Xi’an AMS Center, Institute of Earth Environment, Chinese Academy of Sciences, Xi’an, 710061, China. ^2^ Technical University of Denmark, Center for Nuclear Technologies, Risø Campus, Roskilde, 4000, Denmark. ^3^ Department of Geology, United Arab Emirates University, Al Ain, 17511, United Arab Emirates. ^4^ Department of Earth Sciences, Uppsala University, Uppsala, 75120, Sweden. ^5^ Tandem Laboratory, Uppsala University, Uppsala, 75120, Sweden. ^6^ School of Nuclear Science and Technology, Lanzhou University, Lanzhou, 73000, China. ^7^ College of Hydrology & Water Resources, Hohai University, Nanjing, 210098, China.

**Preparation of ^129^I standards.** Two ^129^I standard solutions, with a total iodine concentration of 1.00 mg/ml and ^129^I/^127^I atomic ratios of 9.954×10^−12^ and 1.138×10^−10^ respectively, were first prepared by dilution of ^129^I standard solution (NIST-SRM-4949c) with ^127^I carrier solution. Two ^129^I working solutions were prepared by mixing the above prepared ^129^I standard solution with NaCl solution in Cl/I mass ratio of 2:1. From each standard, 1.0 ml of working solution was taken to a 15 ml centrifuge tube, 0.5 kBq of ^125^I^-^ tracer, 0.10 ml of 2.0 mol/L NaHSO_3_ and 0.20 mL of 3.0 mol/L HNO_3_, were added and mixed. 0.20 ml of 1.0 mol/L AgNO_3_ was added to coprecipitate iodine as AgI–AgCl after centrifuge, the precipitate was sequentially washed with 3.0 mol/L HNO_3_ and deionized water. For ^129^I standards in AgI form, the ^129^I/^127^I standard solution with a total iodine concentration of 1.0 mg/ml was first converted to iodide by NaHSO_3_ in acidic medium, and then AgNO_3_ was added to directly precipitate iodide as AgI.

**Chemical separation of ^125^IO_3_^-^ tracer.** Iodine in the commercial ^125^I tracer exists as iodide (NaI). To synthesize ^125^IO_3_^-^ tracer, ^125^I^-^ solution was taken to a beaker, NaClO was added, and then HCl is added to adjust pH 1-2 to oxidize iodide to iodate. The remained NaClO in the solution was decomposed by heating at 80℃. The reside is dissolved in water and passed through a small anion exchange column (AG 1×4 resin, NO_3_^−^ form, 1.0 cm in diameter and 5 cm in height). The effluent containing iodate was collected and used a ^125^IO_3_^-^ tracer.

**^127^I^-^ and ^127^IO_3_^-^ preparation for ICP-MS.** 50 ml of seawater was taken to a beaker and ^125^I^−^ was spiked. The sample was loaded to an anion exchange column (1.0 × 5.0 cm, AG1×4 resin, NO_3_^−^ form), the column is rinsed with 10 ml of 0.2 mol/L NaNO_3_. The influent and rinse solution were collected and combined for ^127^IO_3_^-^ determination. Iodide on the column was eluted with 5% NaClO, and the eluate was used for determination of ^127^I^−^. ^125^I in the iodide fraction was measured by gamma spectrometer to monitor chemical recovery of iodide during column separation, which is used to correct the iodide concentration in the seawater.

**Supplementary Tables**

**Table S1** Analytical results of ^127^I and ^129^I species in the Antarctic during the period of November 2010 and January 2011.

**Table S2** Ratios of iodide and iodate (mol/mol) in the Antarctic seawater.

**Table S3** Sampling locations and parameters of Antarctic seawater.

Table S1 Analytical results of ^127^I and ^129^I species in the Antarctic during the period of November 2010 and January 2011.

| Sampling site | ^127^I (μmol/L) | | | ^129^I (×10^6^ atoms/L) | | | ^129^I/^127^I (×10^-11^)  atomic ratio |
| --- | --- | --- | --- | --- | --- | --- | --- |
|  | total iodine | iodide | iodate | total iodine | iodide | iodate |  |
| 1 | 0.45±0.01 | 0.18±0.01 | 0.27±0.01 | 2.65±0.18 | 1.63±0.10 | 1.02±0.09 | 0.98±0.07 |
| 2 | 0.41±0.01 | 0.24±0.01 | 0.18±0.01 | 2.96±0.34 | 1.68±0.12 | 1.28±0.17 | 1.19±0.14 |
| 3 | 0.49±0.02 | 0.20±0.01 | 0.30±0.01 | 2.97±0.22 | 1.98±0.19 | 0.99±0.12 | 1.00±0.07 |
| 4 | 0.44±0.01 | 0.22±0.01 | 0.22±0.01 | 2.53±0.14 | 1.34±0.08 | 1.19±0.09 | 0.96±0.05 |
| 5 | 0.44±0.01 | 0.16±0.01 | 0.28±0.01 | 2.24±0.13 | 1.18±0.07 | 1.06±0.08 | 0.85±0.05 |
| 6 | 0.35±0.01 | 0.23±0.01 | 0.13±0.004 | 1.88±0.10 | 0.88±0.06 | 1.00±0.09 | 0.89±0.05 |
| 7* | 0.35±0.01 | 0.18±0.01 | 0.17±0.01 | 1.51±0.12 | 0.69±0.07 | 0.83±0.10 | 0.71±0.05 |
| 8 | 0.39±0.01 | 0.17±0.01 | 0.22±0.01 | 2.44±0.15 | 1.07±0.06 | 1.38±0.11 | 1.05±0.06 |
| 9 | 0.41±0.01 | 0.25±0.01 | 0.16±0.01 | 2.95±0.14 | 1.45±0.11 | 1.50±0.13 | 1.20±0.06 |
| 10 | 0.33±0.01 | 0.27±0.01 | 0.070±0.002 | 1.82±0.14 | 0.90±0.07 | 0.92±0.10 | 0.96±0.08 |
| 11 | 0.31±0.01 | 0.26±0.01 | 0.050±0.002 | 2.31±0.15 | 0.91±0.06 | 1.40±0.13 | 1.25±0.08 |
| 12 | 0.32±0.01 | 0.25±0.01 | 0.070±0.002 | 2.20±0.16 | 1.47±0.17 | 0.73±0.10 | 1.14±0.08 |
| 13 | 0.31±0.01 | 0.18±0.01 | 0.130±0.004 | 1.69±0.13 | 0.79±0.05 | 0.90±0.09 | 0.90±0.07 |
| 14 | 0.33±0.01 | 0.18±0.01 | 0.140±0.004 | 2.32±0.16 | 1.27±0.11 | 1.05±0.12 | 1.18±0.08 |
| 15 | 0.34±0.01 | 0.24±0.01 | 0.100±0.003 | 1.81±0.35 | 1.28±0.10 | 0.53±0.11 | 0.90±0.17 |
| 16 | 0.45±0.01 | 0.09±0.01 | 0.37±0.01 | 2.08±0.13 | 1.21±0.07 | 0.88±0.07 | 0.76±0.05 |
| 17 | 0.32±0.01 | 0.18±0.01 | 0.140±0.004 | 1.60±0.13 | 0.66±0.05 | 0.94±0.10 | 0.84±0.07 |
| 18 | 0.27±0.01 | 0.17±0.01 | 0.100±0.003 | 2.17±0.14 | 0.91±0.06 | 1.26±0.12 | 1.33±0.09 |
| 19 | 0.29±0.01 | 0.19±0.01 | 0.090±0.003 | 2.37±0.13 | 0.87±0.05 | 1.50±0.11 | 1.37±0.07 |
| 20 | 0.20±0.01 | 0.16±0.01 | 0.040±0.001 | 2.33±0.20 | 0.89±0.06 | 1.43±0.16 | 1.93±0.17 |
| 21 | 0.24±0.01 | 0.18±0.01 | 0.060±0.002 | 2.00±0.13 | 0.76±0.05 | 1.24±0.11 | 1.37±0.09 |
| 22 | 0.30±0.01 | 0.23±0.01 | 0.060±0.002 | 2.42±0.19 | 1.28±0.13 | 1.15±0.15 | 1.35±0.11 |
| 23 | 0.23±0.01 | 0.15±0.01 | 0.080±0.002 | 1.89±0.22 | 0.58±0.04 | 1.31±0.18 | 1.35±0.16 |
| 24 | 0.33±0.01 | 0.18±0.01 | 0.110±0.003 | 2.17±0.25 | 0.73±0.04 | 1.44±0.19 | 1.21±0.14 |
| 25 | 0.36±0.01 | 0.23±0.01 | 0.37±0.01 | 2.75±0.17 | 1.48±0.09 | 1.27±0.11 | 0.76±0.05 |
| 26 | 0.30±0.01 | 0.26±0.01 | 0.070±0.002 | 1.98±0.21 | 1.31±0.14 | 0.67±0.10 | 0.99±0.11 |
| 27 | 0.33±0.01 | 0.080±0.003 | 0.20±0.01 | 1.23±0.14 | 0.45±0.04 | 0.78±0.11 | 0.73±0.08 |
| 28 | 0.60±0.02 | 0.15±0.01 | 0.30±0.01 | 1.98±0.14 | 1.06±0.06 | 0.92±0.08 | 0.74±0.05 |
| 29 | 0.39±0.01 | 0.17±0.01 | 0.22±0.01 | 2.56±0.16 | 1.24±0.11 | 1.32±0.15 | 1.10±0.07 |
| 31 | 0.27±0.01 | 0.18±0.01 | 0.18±0.01 | 3.15±0.18 | 1.72±0.07 | 1.43±0.10 | 1.43±0.08 |
| 32 | 0.33±0.01 | 0.28±0.012 | 0.050±0.002 | 3.10±0.30 | 1.37±0.10 | 1.73±0.21 | 1.55±0.15 |
| 33 | 0.28±0.01 | 0.22±0.01 | 0.120±0.004 | 2.65±0.16 | 1.27±0.08 | 1.39±0.12 | 1.31±0.08 |
| 34 | 0.31±0.01 | 0.12±0.01 | 0.19±0.01 | 2.39±0.19 | 1.22±0.08 | 1.17±0.12 | 1.29±0.10 |
| 35 | 0.29±0.01 | 0.090±0.004 | 0.2±0.01 | 1.64±0.15 | 0.60±0.04 | 1.04±0.12 | 0.93±0.09 |
| 36 | 0.44±0.01 | 0.16±0.01 | 0.120±0.004 | 1.15±0.08 | 0.96±0.09 | 0.19±0.02 | 0.70±0.05 |
| 37* | 0.37±0.01 | 0.11±0.01 | 0.26±0.01 | 1.41±0.11 | 0.96±0.06 | 0.45±0.04 | 0.61±0.05 |
| 38 | 0.30±0.01 | 0.090±0.004 | 0.21±0.01 | 1.28±0.09 | 1.04±0.06 | 0.24±0.02 | 0.70±0.05 |
| 39 | 0.31±0.01 | 0.12±0.01 | 0.19±0.01 | 1.83±0.12 | 1.33±0.11 | 0.50±0.05 | 0.99±0.06 |
| 40 | 0.37±0.01 | 0.050±0.002 | 0.32±0.01 | 3.04±0.30 | 1.11±0.07 | 1.93±0.23 | 1.37±0.14 |
| 41 | 0.34±0.01 | 0.14±0.01 | 0.21±0.01 | 2.80±0.25 | 1.16±0.07 | 1.65±0.17 | 1.35±0.12 |
| 42* | 0.33±0.01 | 0.100±0.004 | 0.23±0.01 | 2.50±0.21 | 1.15±0.06 | 1.35±0.13 | 1.27±0.10 |
| 43 | 0.30±0.01 | 0.050±0.002 | 0.25±0.01 | 1.39±0.16 | 0.51±0.03 | 0.87±0.11 | 0.77±0.09 |
| 44 | 0.39±0.01 | 0.010±0.001 | 0.37±0.02 | 1.54±0.18 | 0.64±0.07 | 0.90±0.14 | 0.66±0.08 |
| 45* | 0.31±0.01 | 0.080±0.003 | 0.23±0.01 | 1.75±0.16 | 0.77±0.04 | 0.98±0.10 | 0.94±0.08 |
| 46 | 0.39±0.01 | 0.11±0.01 | 0.29±0.01 | 2.77±0.24 | 1.36±0.06 | 1.41±0.14 | 1.17±0.10 |
| 47 | 0.23±0.01 | 0.16±0.01 | 0.070±0.002 | 2.32±0.23 | 1.12±0.06 | 1.19±0.13 | 1.70±0.17 |
| 48 | 0.21±0.01 | 0.11±0.01 | 0.090±0.003 | 1.84±0.16 | 1.03±0.07 | 0.81±0.09 | 1.49±0.13 |
| 49 | 0.20±0.01 | 0.11±0.01 | 0.100±0.003 | 1.65±0.18 | 0.76±0.06 | 0.89±0.12 | 1.35±0.14 |
| 50 | 0.24±0.01 | 0.17±0.01 | 0.070±0.002 | 1.98±0.17 | 0.91±0.05 | 1.06±0.11 | 1.35±0.12 |
| 51* | 0.29±0.01 | 0.070±0.003 | 0.22±0.01 | 1.63±0.2 | 1.28±0.07 | 0.35±0.05 | 0.94±0.11 |
| 52 | 0.28±0.01 | 0.11±0.01 | 0.17±0.01 | 1.34±0.13 | 1.01±0.04 | 0.33±0.04 | 0.79±0.08 |
| 53 | 0.27±0.01 | 0.14±0.01 | 0.130±0.004 | 1.91±0.17 | 0.85±0.04 | 1.06±0.11 | 1.17±0.10 |
| 54 | 0.20±0.01 | 0.14±0.01 | 0.050±0.002 | 1.62±0.14 | 0.95±0.05 | 0.67±0.07 | 1.35±0.12 |
| 55 | 0.20±0.01 | 0.17±0.01 | 0.030±0.001 | 2.41±0.15 | 1.20±0.08 | 1.20±0.11 | 1.98±0.12 |
| 56 | 0.34±0.01 | 0.12±0.01 | 0.22±0.01 | 2.75±0.16 | 1.15±0.06 | 1.60±0.12 | 1.35±0.08 |
| 57 | 0.23±0.01 | 0.020±0.001 | 0.22±0.01 | 1.36±0.14 | 0.88±0.06 | 0.47±0.06 | 0.97±0.10 |
| 58 | 0.33±0.01 | 0.17±0.01 | 0.16±0.01 | 1.68±0.12 | 1.39±0.07 | 0.29±0.03 | 0.85±0.06 |
| 59 | 0.34±0.01 | 0.020±0.001 | 0.33±0.01 | 1.49±0.15 | 1.08±0.07 | 0.41±0.05 | 0.72±0.07 |
| 60 | 0.21±0.01 | 0.13±0.01 | 0.080±0.002 | 2.17±0.14 | 1.16±0.05 | 1.00±0.08 | 1.54±0.10 |
| 61 | 0.2±0.01 | 0.080±0.003 | 0.130±0.004 | 1.69±0.12 | 1.08±0.06 | 0.61±0.05 | 1.38±0.10 |
| 62 | 0.27±0.01 | 0.100±0.004 | 0.17±0.01 | 2.20±0.26 | 0.97±0.06 | 1.23±0.17 | 1.37±0.16 |
| 63 | 0.35±0.01 | 0.080±0.003 | 0.28±0.01 | 2.69±0.16 | 1.59±0.12 | 1.10±0.11 | 1.27±0.07 |
| 64 | 0.24±0.01 | 0.17±0.01 | 0.070±0.002 | 2.61±0.17 | 1.58±0.08 | 1.03±0.09 | 1.78±0.12 |
| 65 | 0.34±0.01 | 0.18±0.01 | 0.16±0.01 | 2.57±0.20 | 1.63±0.12 | 0.95±0.10 | 1.24±0.10 |

* These data have been reported in the Reference 23.

Table S2 Ratios of iodide and iodate (mol/mol) in the Antarctic seawater.

| sampling site | ^127^I^-^/^127^IO_3_^-^ | ^129^I^-^/^129^IO_3_^-^ |
| --- | --- | --- |
| 1 | 0.69±0.03 | 1.59±0.17 |
| 2 | 1.34±0.06 | 1.32±0.20 |
| 3 | 0.65±0.03 | 2.01±0.31 |
| 4 | 1.03±0.04 | 1.12±0.11 |
| 5 | 0.56±0.02 | 1.12±0.11 |
| 6 | 1.78±0.08 | 0.88±0.10 |
| 7 | 1.05±0.05 | 0.83±0.13 |
| 8 | 0.75±0.03 | 0.77±0.08 |
| 9 | 1.63±0.07 | 0.97±0.11 |
| 10 | 3.95±0.17 | 0.97±0.13 |
| 11 | 4.97±0.21 | 0.65±0.08 |
| 12 | 3.43±0.15 | 2.02±0.35 |
| 13 | 1.48±0.06 | 0.88±0.10 |
| 14 | 1.26±0.05 | 1.21±0.17 |
| 15 | 2.4±0.10 | 2.40±0.54 |
| 16 | 0.24±0.01 | 1.37±0.14 |
| 17 | 1.32±0.06 | 0.69±0.09 |
| 18 | 1.78±0.08 | 0.73±0.08 |
| 19 | 2.03±0.09 | 0.58±0.05 |
| 20 | 3.70±0.16 | 0.62±0.08 |
| 21 | 2.81±0.12 | 0.61±0.07 |
| 22 | 3.76±0.16 | 1.11±0.18 |
| 23 | 1.98±0.08 | 0.44±0.07 |
| 24 | 1.62±0.07 | 0.51±0.07 |
| 25 | 0.61±0.03 | 1.17±0.12 |
| 26 | 3.51±0.15 | 1.97±0.36 |
| 27 | 0.38±0.02 | 0.58±0.10 |
| 28 | 0.48±0.02 | 1.16±0.12 |
| 29 | 0.76±0.03 | 0.93±0.14 |
| 31 | 1.02±0.04 | 1.21±0.10 |
| 32 | 5.85±0.24 | 0.79±0.11 |
| 33 | 1.8±0.08 | 0.91±0.10 |
| 34 | 0.64±0.03 | 1.05±0.12 |
| 35 | 0.48±0.02 | 0.58±0.07 |
| 36 | 1.37±0.06 | 5.19±0.77 |
| 37 | 0.43±0.02 | 2.14±0.24 |
| 38 | 0.42±0.02 | 4.26±0.44 |
| 39 | 0.62±0.03 | 2.64±0.34 |
| 40 | 0.15±0.01 | 0.58±0.08 |
| 41 | 0.68±0.03 | 0.70±0.09 |
| 42 | 0.42±0.02 | 0.85±0.09 |
| 43 | 0.21±0.01 | 0.59±0.08 |
| 44 | 0.030±0.003 | 0.71±0.13 |
| 45 | 0.35±0.02 | 0.79±0.09 |
| 46 | 0.37±0.02 | 0.96±0.11 |
| 47 | 2.39±0.10 | 0.94±0.11 |
| 48 | 1.19±0.05 | 1.27±0.16 |
| 49 | 1.08±0.05 | 0.86±0.13 |
| 50 | 2.60±0.11 | 0.86±0.10 |
| 51 | 0.33±0.01 | 3.67±0.54 |
| 52 | 0.67±0.03 | 3.07±0.36 |
| 53 | 1.02±0.04 | 0.81±0.09 |
| 54 | 2.70±0.12 | 1.41±0.16 |
| 55 | 4.85±0.21 | 1.00±0.11 |
| 56 | 0.53±0.02 | 0.72±0.06 |
| 57 | 0.070±0.004 | 1.87±0.26 |
| 58 | 1.04±0.04 | 4.81±0.50 |
| 59 | 0.050±0.003 | 2.67±0.37 |
| 60 | 1.58±0.07 | 1.16±0.10 |
| 61 | 0.62±0.03 | 1.76±0.18 |
| 62 | 0.56±0.02 | 0.79±0.12 |
| 63 | 0.27±0.01 | 1.45±0.18 |
| 64 | 2.39±0.10 | 1.53±0.15 |
| 65 | 1.16±0.05 | 1.72±0.22 |

Table S3 Sampling locations and parameters of Antarctic seawater.

| Sampling site | longitude | latitude | Salinity (psu) | Temperature  (ºC) | chlorophyll (μg/L) | pCO_2_ (μatm) |
| --- | --- | --- | --- | --- | --- | --- |
| 1 | 67.42ºW | 59.77ºS | 34.0 | 1.0 | 0.29 | ­ |
| 2 | 67.96ºW | 60.73ºS | 34.0 | 3.1 | 0.60 | 360 |
| 3 | 68.41ºW | 61.52ºS | 34.0 | 1.1 | 1.10 | 223 |
| 4 | 69.46ºW | 63.25ºS | 34.0 | -0.1 | 0.78 | 349 |
| 5 | 70.01ºW | 64.16ºS | 34.0 | -0.4 | 1.60 | 235 |
| 6 | 71.48ºW | 65.09ºS | 34.0 | -0.5 | 3.50 | 310 |
| 7 | 71.60ºW | 66.36ºS | 34.0 | -0.5 | 1.00 | 287 |
| 8 | 73.02ºW | 66.87ºS | 34.0 | -0.5 | 1.20 | 290 |
| 9 | 75.71ºW | 66.85ºS | 34.0 | -0.7 | 1.05 | 326 |
| 10 | 78.76ºW | 66.73ºS | 34.0 | -0.4 | 3.60 | 290 |
| 11 | 82.31ºW | 66.22ºS | 34.0 | -0.6 | 1.20 | 335 |
| 12 | 84.56ºW | 66.05ºS | 33.0 | -1.1 | 0.67 | 325 |
| 13 | 86.99ºW | 66.57ºS | 33.1 | -0.8 | 1.02 | 330 |
| 14 | 90.33ºW | 66.66ºS | 33.5 | -1.4 | 0.51 | 328 |
| 15 | 92.77ºW | 66.87ºS | 33.6 | -1.5 | 0.69 | 343 |
| 16 | 93.37ºW | 67.03ºS | 33.6 | -1.5 | 2.34 | 344 |
| 17 | 95.92ºW | 66.91ºS | 33.6 | -1.4 | 0.42 | 337 |
| 18 | 97.82ºW | 66.84ºS | 33.6 | -1.6 | 0.80 | 334 |
| 19 | 100.38ºW | 66.70ºS | 33.6 | -1.6 | 1.23 | 339 |
| 20 | 103.28ºW | 66.85ºS | 33.7 | -1.6 | 0.91 | 354 |
| 21 | 104.45ºW | 66.99ºS | 33.7 | -1.6 | 0.76 | 362 |
| 22 | 106.40ºW | 67.63ºS | 33.7 | -1.5 | 1.59 | 358 |
| 23 | 108.45ºW | 68.53ºS | 33.8 | -1.6 | 0.24 | 396 |
| 24 | 110.27ºW | 68.98ºS | 33.9 | -1.5 | 0.18 | 411 |
| 25 | 113.19ºW | 69.40ºS | 33.9 | -1.6 | 0.40 | 408 |
| 26 | 114.88ºW | 70.22ºS | 33.9 | -1.6 | 0.28 | 415 |
| 27 | 115.16ºW | 70.96ºS | 33.9 | -1.6 | 0.18 | 441 |
| 28 | 117.30ºW | 71.64ºS | 33.9 | -1.6 | 0.41 | 423 |
| 29 | 113.25ºW | 74.19ºS | 33.8 | -1.3 | 0.26 | 477 |
| 31 | 109.49ºW | 73.26ºS | 33.2 | -1.6 | 0.69 | 404 |
| 32 | 108.65ºW | 73.37ºS | 33.6 | -1.6 | 0.49 | 415 |
| 33 | 112.65ºW | 73.56ºS | 33.8 | 0.0 | 26.90 | 163 |
| 34 | 116.30ºW | 72.72ºS | 33.5 | -1.6 | 2.83 | 259 |
| 35 | 116.90ºW | 72.03ºS | 33.2 | -1.3 | 3.00 | 237 |
| 36 | 114.57ºW | 71.55ºS | 33.7 | -1.7 | 0.54 | 406 |
| 37 | 118.27ºW | 71.85ºS | 33.4 | -1.1 | 1.96 | 333 |
| 38 | 118.86ºW | 71.80ºS | 33.5 | -1.0 | 2.70 | 327 |
| 39 | 121.81ºW | 71.78ºS | 33.4 | -1.0 | 0.54 | 371 |
| 40 | 126.45ºW | 71.18ºS | 33.3 | -1.2 | 0.97 | 370 |
| 41 | 129.80ºW | 71.10ºS | 33.3 | -1.1 | 1.72 | 365 |
| 42 | 134.80ºW | 71.70ºS | 33.2 | -1.2 | 0.64 | 376 |
| 43 | 136.91ºW | 72.13ºS | 33.2 | -0.7 | 0.86 | 369 |
| 44 | 138.21ºW | 71.21ºS | 33.2 | -0.8 | 0.78 | 371 |
| 45 | 139.15ºW | 72.33ºS | 33.3 | -1.2 | 0.90 | 378 |
| 46 | 143.10ºW | 72.85ºS | 33.2 | -1.2 | 0.58 | 364 |
| 47 | 144.42ºW | 73.29ºS | 33.1 | -1.3 | 0.55 | 353 |
| 48 | 145.62ºW | 73.57ºS | 33.2 | -1.5 | 1.12 | 364 |
| 49 | 147.48ºW | 73.95ºS | 33.2 | -0.4 | 2.40 | 332 |
| 50 | 149.19ºW | 74.06ºS | 33.2 | 0.3 | 5.70 | ­ |
| 51 | 150.62ºW | 74.25ºS | 33.8 | 0.6 | 6.90 | ­ |
| 52 | 159.66ºW | 74.99ºS | 33.9 | -0.2 | 5.45 | 211 |
| 53 | 161.39ºW | 75.10ºS | 33.9 | 0.3 | 7.10 | 181 |
| 54 | 163.56ºW | 75.26ºS | 33.9 | 0.2 | 18.70 | 181 |
| 55 | 167.01ºW | 75.51ºS | 33.9 | 0.0 | 16.40 | 202 |
| 56 | 173.70ºW | 75.99ºS | 33.9 | -0.6 | 0.73 | 366 |
| 57 | 174.89ºW | 76.08ºS | 34.0 | -0.6 | 0.78 | 373 |
| 58 | 179.79ºW | 76.41ºS | 34.2 | -0.4 | 1.66 | 271 |
| 59 | 177.62ºE | 76.62ºS | 34.3 | -0.5 | 2.67 | 242 |
| 60 | 175.76ºE | 76.76ºS | 34.3 | -0.3 | 2.20 | 242 |
| 61 | 174.48ºE | 76.85ºS | 34.3 | -0.2 | 2.20 | 223 |
| 62 | 173.24ºE | 76.92ºS | 34.3 | -0.2 | 2.20 | 230 |
| 63 | 169.02ºE | 77.05ºS | 33.9 | -0.5 | 2.36 | 215 |
| 64 | 168.85ºE | 77.14ºS | 34.0 | -0.3 | 2.39 | 214 |
| 65 | 168.34ºE | 77.17ºS | 34.2 | 0.1 | 2.41 | 221 |

**Supplementary Figures**

**Figure S1** Variation of ^129^I/^127^I in surface seawater in the Antarctic.

**Figure S2** Distribution of temperature, chlorophyll and pCO_2_ in the surface seawater. The original map was constructed by a free software Ocean Date View (ODV 4.7.8) (Schlitzer, R., Ocean Data View, odv.awi.de, 2017).

**Figure S3** Schematic diagram of the analytical procedure for speciation analysis of ^129^I in seawater.


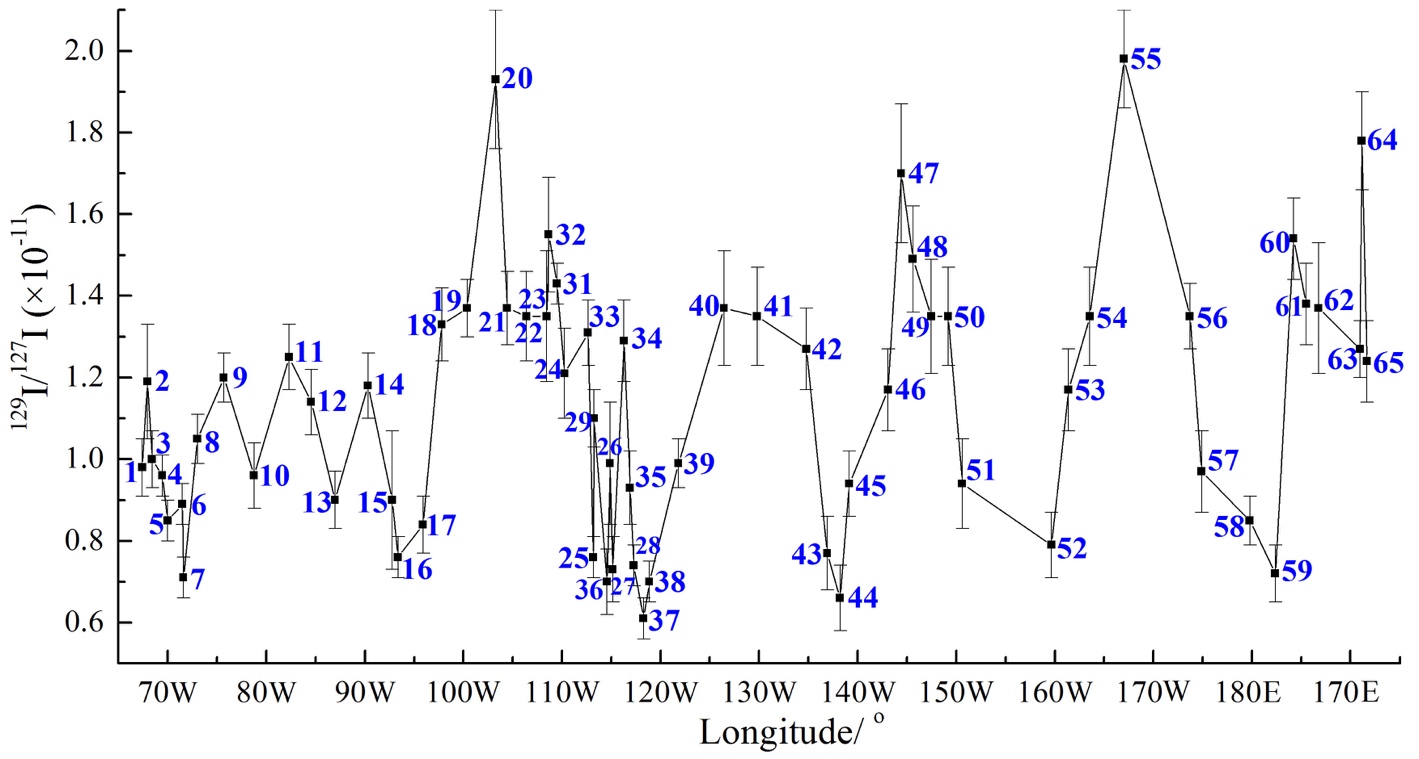


Figure S1 Variation of ^129^I/^127^I in surface seawater in the Antarctic.


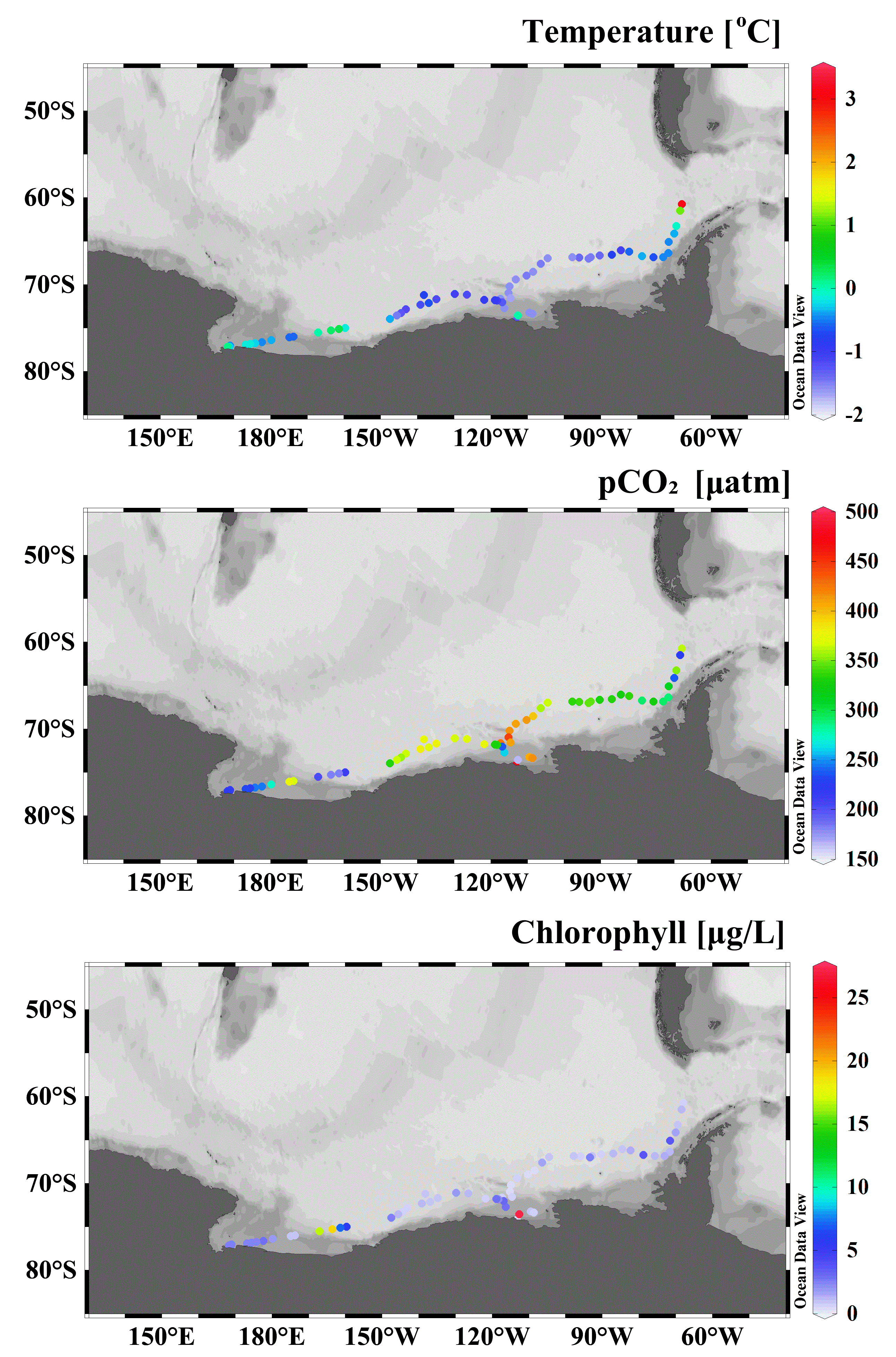


Figure S2 Distribution of temperature, chlorophyll and pCO_2_ in the surface seawater. The original map was constructed by a free software Ocean Date View (ODV 4.7.8) (Schlitzer, R., Ocean Data View, odv.awi.de, 2017).


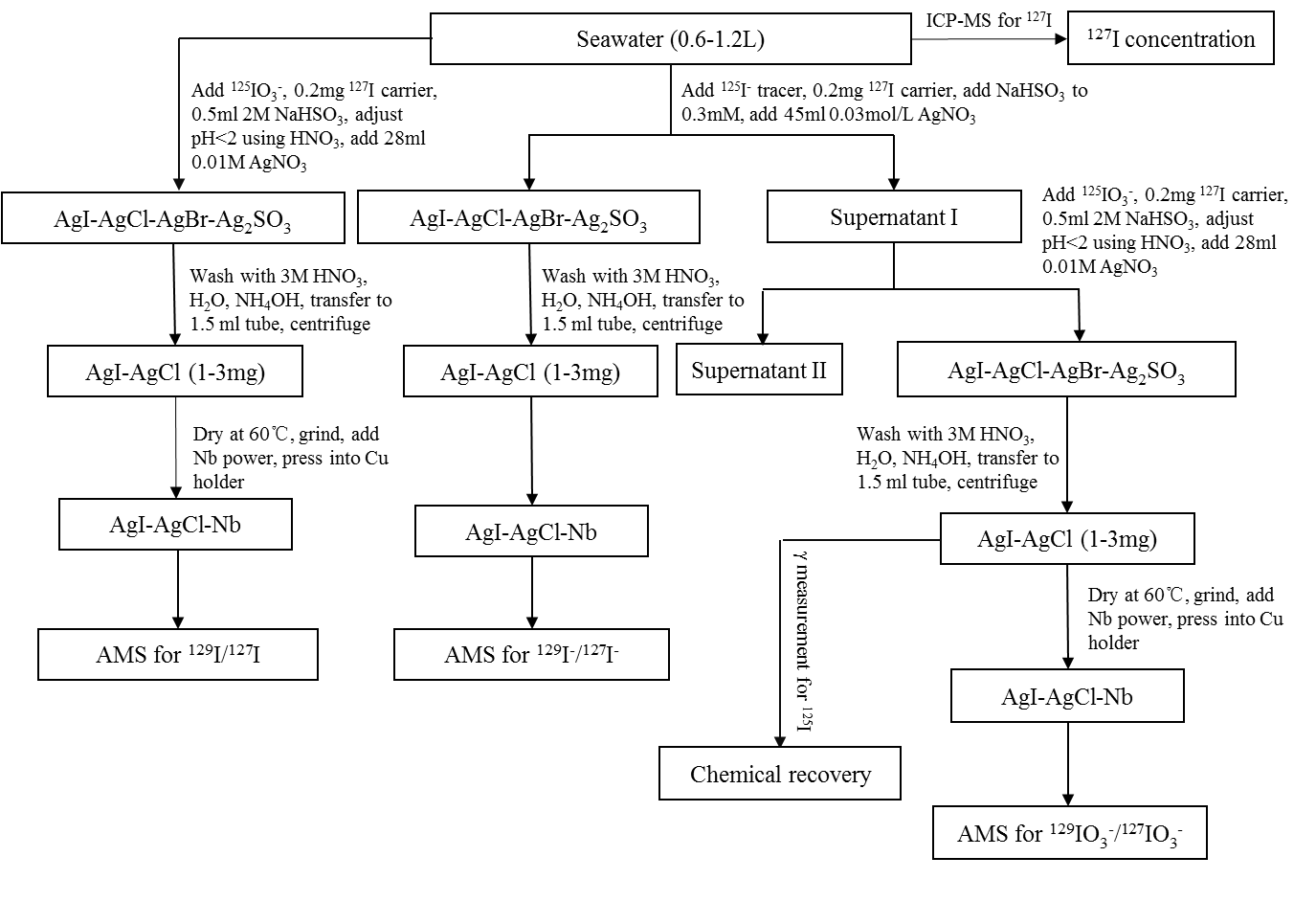


Figure S3 Schematic diagram of the analytical procedure for speciation analysis of ^129^I in seawater.
